# Lentinula edodes β-glucan enriched diet induces pro- and anti-inflammatory macrophages in rabbit

**DOI:** 10.1080/16546628.2017.1412791

**Published:** 2017-12-12

**Authors:** Helena Crespo, Hugo Guillén, Lorena de Pablo-Maiso, Carmen Gómez-Arrebola, Gregorio Rodríguez, Idoia Glaria, Damián de Andrés, Ramsés Reina

**Affiliations:** ^a^ Department of Animal Health, Instituto de Agrobiotecnología (UPNA-CSIC-Gob. de Navarra; IdAB), Navarra, Spain; ^b^ Glucanfeed, Calahorra, Spain; ^c^ Cunicultura Villamalea, Albacete, Spain

**Keywords:** Fungal β-Glucan, macrophages, polarization, rabbit, diet

## Abstract

β-glucans exhibited in cell walls of several pathogens as bacteria or fungi are sensed by pathogen recognition receptors such as scavenger receptors present in antigen presenting cells, i.e., macrophages. β-glucans obtained from Shiitake mushrooms were chemically characterized. A β-glucan supplemented diet was assayed for 30 days in rabbits aiming to characterize the immune response elicited in blood-derived macrophages. M1 and M2 profiles of macrophage differentiation were confirmed in rabbits by *in vitro* stimulation with IFN-γ and IL-4 and marker quantification of each differentiation pathway. Blood derived macrophages from rabbits administered *in vivo* with the β-glucan supplemented diet showed higher IL-4, IFN-γ and RAGE together with lower IL-10 relative expression, indicative of an ongoing immune response. Differences in IL-1β, IL-13 and IL-4 expression were also found in rabbit sera by ELISA suggesting further stimulation of the adaptive response. Recent challenges in the rabbit industry include the search of diet supplements able to elicit an immune stimulation with particular interest in facing pathogens such as viruses or bacteria. β–glucans from fungi may contribute to maintain an immune steady state favouring protection and thus reducing antibiotic treatment.

## Introduction

The ability of β-glucans to stimulate the immune system and to modulate pathogenic processes is known since more than 50 years. Founder experiments were based on experimental observations, but since a decade mechanistic insights are being unveiled [1]. However, results obtained are often controversial mainly due to inconsistent comparisons between uncharacterised β-glucan preparations, from different origins (fungi, cereals) or with different molecular weight. These discrepancies could be attributed to the different ability of β-glucans to stimulate the immune system, which is highly dependent on the ramification rate, length and tertiary structure.

Among mushrooms Shiitake (*Lentinula edodes*) produces high quantities of branched β (1–3)(1–6)-D-glucan (Lentinan) [2,3]. Interaction of β-glucans with different cellular receptors inducing intracellular signalling may activate different profiles of the immune response [1,4,5].

Macrophages are key cells in orchestrating pro- and anti-inflammatory responses being integral part of the innate and the adaptive immune response. M1, or classically activated macrophages, produce high levels of pro-inflammatory cytokines and M2, or alternatively activated macrophages, show an anti-inflammatory profile involved in wound healing and tissue repair [6,7]. Molecules differentially expressed in each macrophage subpopulation can be employed as markers of a specific differentiation pathway [8] also in rabbits [9].

*In vivo* administration of β-glucans has been beneficial against pathogens such as fungi, bacteria, viruses and protozoa [10] in several animal models by stimulating innate immunity, cytokine production and oxidative stress. Despite this proinflammatory role, β-glucans may decrease tissue damage in already established inflammatory processes [11].

In cell culture, Lentinan from Shiitake induced altered cytokine production, augmented cytotoxicity against cancer cells and also anti-inflammatory protective responses [12–15].

The European rabbit industry is mainly based in France, Italy and Spain. One of the bottlenecks faced by the rabbit industry is the reduction in the use of antibiotics. European legislation claims for a drastic reduction of antibiotics in animal production, favouring new approaches that sustain strong immune responses against breeding stresses. Thus, the administration of β-glucans may be useful in animal industries that require reduction of antibiotic treatments but maintaining an alert immune status to efficiently face pathogens, such as rabbits.

This work aims at characterizing the macrophage differentiation in production rabbits submitted to a β-glucan enriched diet. Results show a clear immune activation after intake of β-glucans from Shiitake. This immune activation may protect rabbits from infectious agents in specific immunosuppressed physiological situations, such as weaning or at the perinatal stage, thereby potentially reducing antibiotic administration.

## Material and methods

### Shiitake production and β-glucans purification

Shiitake mushrooms were purchased from Vallondo C.B. (Autol, La Rioja, Spain), dehydrated through oven heating with forced ventilation always at temperatures under 60°C for less than 10 hours. Subsequently the dried samples were milled, mixed and sieved (particle size under 2 mm). Extraction of β-glucans was performed following previously described procedures [16]. Briefly, raw material was resuspended in deionized water and the mixture was heated to 100–110ºC with stirring for 45 minutes. Subsequently 80% cold ethanol was added and stirring was maintained for 30 minutes. The mixture was kept at 5ºC overnight without stirring. The obtained mixture was filtered, dried and milled (particle size under 0.5 mm).

### β-glucans quantification

β-glucan content of the purified product was evaluated with the Assay for mushroom and yeast β-glucans determination (Megazyme™). In a first step, total glucans (including D-glucose oligosaccharides, sucrose and D-glucose free) were quantified and after the conditions were changed, another test quantifies the α-glucans content (including starch, glycogen and the D-glucose and D-glucose sucrose-free quantification) within the same sample. β-glucan quantification is then obtained by the subtraction of these two determinations.

Quantification of (1,3→1,6)-β-glucan content in Shiitake extracts was performed by the acid/basic fractions method described by Mölleken et al. [2] with slight modifications. Briefly, samples were resuspended in citric buffer solution (0.5M NaOH, KOH 1M 6M HCl, 1M HCl, 0.55 M HCl, 6M KOH buffer 0.2M citric acid/NaOH pH = 7.00) and staining solution (0.08 grams of Congo Red in 100ml of citric buffer). A standard curve of schizophyllan, a lentinan from *Schizophyllum commune* (0–50 μg/ml), was prepared thereby lentinan content in samples was inferred.

### Animals and samples

Animal procedures were described in the project approved by the Ministry of agriculture, food and environment (MAGRAMA: 20140020001815).

One hundred HYPLUS hybrid crossbreed Californian and New Zealand rabbits of 45 days of age (Grimaud Fréres, La Corbiere, France) were selected from the same farm and divided in two groups (50 each). One group was fed with a conventional diet including proteins, lipids, dietary fiber, ashes, calcium, phosphate and sodium in the presence of oxitetracyclin, neomycin sulfate and valnemulin hydrochloride; the other group was fed with the same conventional diet supplemented with 5% of purified β-glucans extracted from Shiitake for a 30 day-period. Blood samples were collected by intracardiac puncture before the differential feeding (time 0) and at 30 days (time 1) in heparin tubes (Vacutainer). Sera were stored at −20ºC for ELISA determinations.

Blood monocyte-derived macrophages (BDM) were obtained at days 0 and 30 by adherence of peripheral blood monocytes after Lymphoprep gradient centrifugation (δ = 1.077; Asix-Shield, Oslo). BDM were cultured in RPMI medium supplemented with 10 mM sodium pyruvate, 1% non-essential amino acids, 1% vitamins, 1% antibiotics/antimycotics mix and 1:1000 gentamicin, 1% L-glutamine and 50 μM 2-mercaptoethanol (Sigma) for selection of adherent cells during 2 h. Cells were washed three times with ice-cold PBS and washes containing non-adherent cells were also collected. Medium was replaced with fresh RPMI further supplemented with 10% foetal bovine serum and cells incubated for 6 days. Non-adherent cells containing lymphocytes were seeded at a density of 10^5^ cells/well in 96-well round bottom plates and incubated with 1µg/well of the mitogen Concanavalin A (ConA; Sigma) for 48 hours. Plates were centrifuged and supernatants collected for cytokine assessment by ELISA.

### RNA extraction and retrotranscription

6 days-cultured BDM were washed and collected in TRI Reagent® (Ambion) for RNA extraction. Total RNA isolation was performed by chloroform extraction and isopropanol precipitation. RNA was treated with TURBO DNAse™ (Ambion) and purified by extraction with phenol acid, chloroform and ethanol precipitation. One µg of total RNA was retrotranscribed to cDNA using Transcriptor First Strand cDNA synthesis kit (Roche) following manufacturers protocol with oligo-dT primers.

### Blood derived macrophages stimulation

BDM were obtained from healthy (n = 7) rabbits and were cultured in the presence of IFN-γ and IL-4 at 50 µg/ml [17]. After two rounds of stimulation, 3 days each, BDM and supernatants were collected for relative expression profile evaluation and for cytokine measurements by ELISA respectively.

### Relative expression of macrophage differentiation markers by real time qPCR

Relative expression of pro- and anti-inflammatory cytokines as well as membrane receptors differentially associated to macrophage maturation into M1/M2 profiles, including IL-4, IL-10, IL-6, TNF-α, IL-1β, IFN-γ, mannose receptor (MR) and the receptor for advanced glycosilation end-products (RAGE or AGER) was evaluated in all the animals. Quantitative RT-PCR was performed with SYBR mix (Takara) using ten-fold diluted cDNA from BDMs as template. Specific primers for amplification of IL-4, IL-10, IFN-γ, IL-6, TNF-α, IL-1β and RAGE were described previously [18,19]. In the case of MR, amplification primers were designed based on rabbit sequences available at NCBI Genbank (NC_013684.1) being primer forward 5ʹAAATGTTGAATTTTGTGGTGAGCTA3ʹ and reverse 5ʹTGGCAAATCCAGTTGTTAAGGTGTT3ʹ. Amplification was performed in an ABI Prism 7900HT detector system (Applied Biosystems) following 45 cycles at 95 ºC for 30 s and 60 ºC for 1 min and a dissociation curve analysis was carried out to verify the specificity of the product.

GAPDH expression was included as housekeeping gene using primers described previously [20]. GAPDH Ct values were subtracted from the Ct values obtained for each marker molecule in order to obtain the ΔCt used for comparisons.

Standard curves of each PCR reaction were constructed by amplifying 10-fold dilutions of plasmids containing each of the amplicons. Slopes, PCR efficiencies (E = 10^–1/slope^) and correlation coefficients (R^2^ value) for all standard curves were analyzed in order to validate the qPCR results.

### Cytokine measurements by ELISA

Non-adherent cells were seeded at 10^5^ cells/well in 96-well round bottom plates and incubated for 48 h with 1µg/well of the mitogen Concanavalin A (ConA; SIGMA). IL1-β, TNF-α, IL-12, IL-13 and IL-4 were measured in supernatants with commercial ELISA kits (Cusabio Biotech Co.) following manufacturer’s protocol. Absorbance values obtained after 30 days of supplemented diet were normalized with pre-diet values and compared between groups.

Supernatants from BDMs stimulated with IFN-ɣ or IL-4 were also submitted to these ELISA kits.

IFN-ɣ and IL-4 were measured in sera collected at time 0 and after 30 days from rabbits fed with β-glucans enriched diet (n = 12) and normal diet (n = 4) following manufacturer’s instructions (Cusabio Biotech Co.).

### Statistical analysis

Gene expression data were analysed using the REST 2009 software [21] and the provided *p* value used for the likelihood of up-regulation or down-regulation of each gene between treated and control groups.

Absorbances (O.D. 450nm) obtained in supernatants after macrophage differentiation were compared with Mann-Whitney-U test for non-related samples. Absorbance values obtained in control and Shiitake groups after 30 days of respective treatment were subtracted from values recorded at the beginning of the experiment (Δ Absorbance). Δ Absorbance values were used for comparisons with Mann-Whitney-U test for non-related samples.

## Results

### M1/M2 differentiation in rabbits

BDM from healthy rabbits were obtained and stimulated *in vitro* with IFN-ɣ and IL-4 and the relative expression of M1 proinflammatory markers (IL-6, TNF-α, IL-1β, IFN-ɣ and RAGE) as well as M2 markers (MR, IL-4 and IL-10) was quantified.

**Figure 1.**
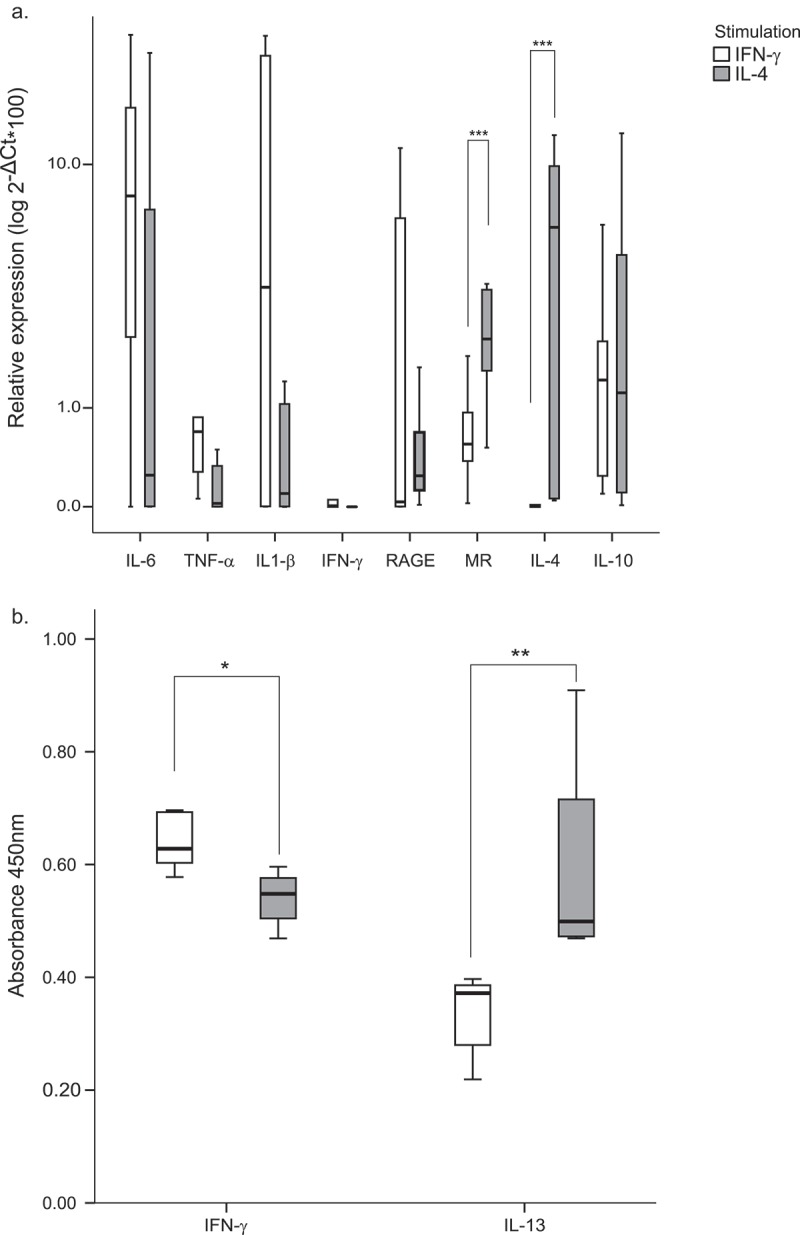
**BDM *in vitro* differentiation with M1/M2 hallmark cytokines**. BDM were cultured in the presence of the cytokines IFN-ɣ or IL-4. (a) Relative expression of M1 (IL-6, TNFα, IL1-β, IFN-ɣ and RAGE) and M2 (MR, IL-4, IL-10) markers was measured by quantitative RT-PCR. The values are expressed as the mean of 2-ΔCt*100 (±SE) in logarithm scale of at least 3 independent experiments normalized to the endogenous gene GAPDH. (b) Cytokine production in supernatants was assessed by rabbit IFN-ɣ and IL-13 ELISA kits. Values are represented as the median of the absorbance at 450nm (± interquartile range). * p < 0.05; ** p < 0.01; *** p < 0.001.

IFN-ɣ and IL-4 stimulated macrophages from rabbits showed typical markers of pro- and anti-inflammatory differentiation pathways respectively. Comparative analysis indicated that IFN-γ stimulated macrophages significantly increased levels of the proinflammatory marker TNF-α (p = 0.033) and decreased levels of MR and IL-4 (Figure 1). IL-6, IL-1β and IFN-γ showed non-significant increases in IFN-ɣ stimulated macrophages (Figure 1(a)).

To further characterize the *in vitro* differentiation, IFN-ɣ and IL-13 production was also measured in stimulated BDMs supernatants by ELISA (Figure 1(b)). IFN-ɣ stimulated BDM showed non-significant increased levels of IFN-ɣ production (p = 0.19) while IL-4 stimulated cultures produced higher amounts of IL-13 in the supernatants (p = 0.010), confirming the relative expression data at the RNA level.

### β-glucans from shiitake are immunostimulatory through diet supplementation

**Figure 2.**
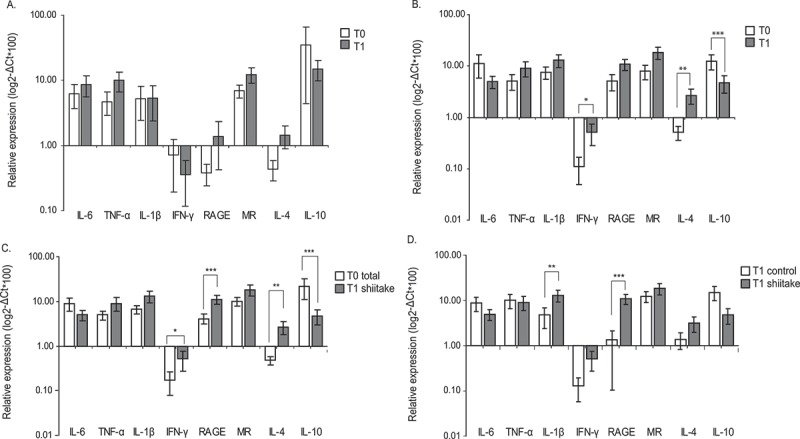
β-glucans from shiitake modulate BDM immune responses in rabbits after supplemented diet. Relative expression of M1/M2 markers was determined by quantitative RT-PCR in BDM obtained from animals following conventional diet (control group, A) or enriched diet (shiitake group, B) at time 0 (T0) and time 1 (30 days of supplemented diet, T1). Animals at time 0 were pooled (T0 total) and compared with treated animals at time 1 (C). Control and shiitake groups were also compared after 30 days of the specific diet (D). Values represented are the mean of 2-ΔCt*100 (±SE) in logarithm scale. Statistical analysis used for comparisons were performed with REST 2009 software (Qiagen). * p < 0.05; ** p < 0.01; *** p < 0.001.

Control animals fed during 30 days with normal diet did not show changes in the relative expression of any marker assayed indicating no immunological stimulation due to uncontrolled environmental factors (Figure 2(a)).

Instead, animals fed with β-glucans showed a clear increase in the relative expression of IL-4 and IFN-γ when comparing values with those obtained at the beginning of the experiment. Interestingly, this was accompanied by a drastic decrease in IL-10 expression (Figure 2(b)). IL-1β and RAGE showed an increased expression of 2.75 and 2.66-fold respectively after diet supplementation, however none of them was statistically different from time zero (p = 0.066 and 0.1 respectively; Figure 2(b)).

Comparing relative expression levels from all the animals involved in the experiment pooled at time zero and those obtained after the consumption of the supplemented diet, raised significant increases in IL-4, IFN-γ and RAGE as well as a reduced IL-10 production (Figure 2(c)). Interestingly, a trend to increased IL-1β levels was found (p = 0.065).

Direct comparison between both groups after 30 days of normal or supplemented diet confirmed the increase in IL-1β transcripts, as well as the increased RAGE production in supplemented animals compared with controls (Figure 2(d)).

### Cytokine production in conA stimulated lymphocytes

**Figure 3.**
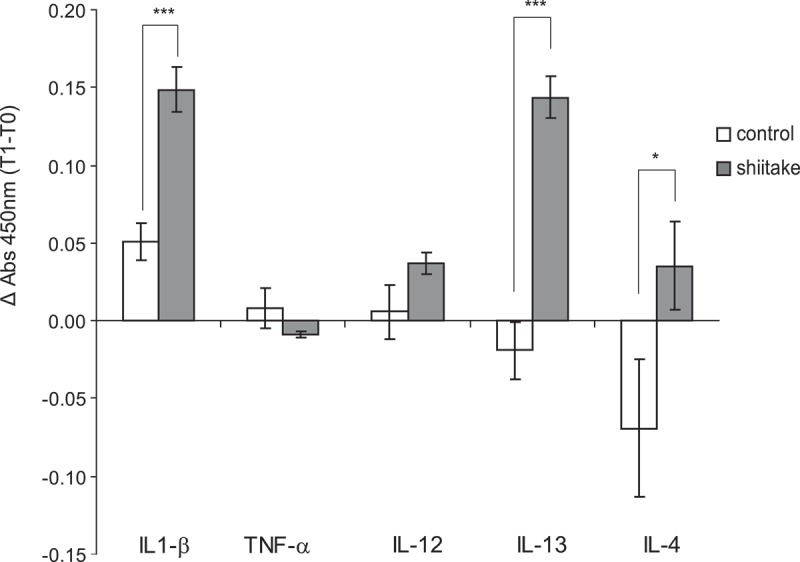
β-glucan supplemented diet elicit cytokine production by ConA stimulated lymphocytes. Cytokine production (IL1-β, TNF-α, IL-12, IL-13 and IL-4) in supernatants from Con A treated lymphocytes was quantified with commercial ELISA kits. Absorbance at 450nm was measured and Δ Absorbance data (time 1 minus time 0) were used for comparisons among groups.

To further investigate the immune responses elicited after β-glucan supplementation, cytokine production in supernatants of ConA stimulated lymphocytes was determined by ELISA, and the increase in absorbance during the experiment (Δ Absorbance) was compared among the two experimental groups. Significant increases were observed in the production of IL1-β, IL-13 and IL-4 (p < 0.001 for IL1-β and IL-13 and p < 0.05 for IL-4) in treated animals compared with the untreated group (Figure 3). IL-12 was also increased while TNF-α values decreased although differences were not significant (p = 0.352 and p = 0.476, respectively).

### Cytokine production in serum

IL-4 production was close to 1.5-fold higher in treated animals, but differences were not significant (p = 0.770). IFN-γ production was not detected in serum samples (data not shown).

## Discussion

Highly branched β-glucans with triple β-helix produced by yeast and fungi have been widely studied as inducers of immune activation which may in turn protect against many types of infection [5]. Despite this, studies unveiling immune mechanisms of this protective role are scarce.

In this randomized double-blinded placebo-controlled study, we aimed to characterize macrophage maturation profile in rabbits fed with a fungi-derived β-glucan supplemented diet. First, we checked the macrophage polarization paradigm in rabbits by stimulating blood-derived macrophages with cytokines inducing the M1 (IFN-γ) or the M2 (IL-4) profiles. Rabbit macrophages have been polarized into M1 and M2 by stimulation with human GM-CSF or M-CSF, respectively [9]. Relative expression of biological markers representative of each maturation state points out for a conserved mechanism among humans, mice, sheep and rabbits generalizing M1 and M2 differentiation pathways. As described, expression of TNF-α and IL-1β stood out in M1 profile while induction of MR and IL-4 were indicative of M2 differentiation. Cytokine production in BDM culture supernatants confirmed mRNA relative quantification results with high production of IFN-ɣ and low IL-13 in M1 macrophages; and low IFN-ɣ and high IL-13 in M2 BDMs.

Supplementation of rabbits’ diet with Shiitake-derived β-glucans for 30 days induced a lower IL-10 expression and higher levels of IL-4 and IFN-γ all of which indicates an ongoing immune response. Further comparisons with the control group also revealed IL1-β and RAGE as significantly induced by the supplemented diet (Figure 2(c,d)). Increased IL-1β, IL-4 or IL-13 cytokine production was observed in the supernatant of ConA-stimulated lymphocytes confirming RNA relative expression results.

β-glucans orally consumed were able to modify significantly the macrophage maturation profile suggesting that protective roles could be based on macrophage differentiation upon recognition of branched β-glucans.

Generally, high molecular weight or highly branched β-glucans (like zymosan), may directly activate phagocytosis and cytotoxicity through oxygen and nitrogen reactive species production, together with the production of IL-8, IL-1β, IL-6 and TNF-α proinflammatory cytokines. β-glucan from *L. edodes* is highly branched and production is high when compared to other mushrooms [2].

β-glucans biological activity may be favoured in mammals by their augmented half-life in the body due to the absence of β-glucanases, so that oxidative processes are involved in β-glucan metabolism [22]. Oral administration of *Aureobasidium pullulans* enriched with branched β-glucans protected mice against a lethal challenge with influenza virus likely driven by the overexpression of retinoic acid-inducible gene I (RIG-I) and melanoma differentiation-associated protein 5 (MDA5) in macrophages linking virus sensor expression with influenza infection prevention [23]. Whether these immunological pathways are also stimulated in ruminants after β-glucans treatment is unknown.

In line with our results, THP1-macrophages stimulated *in vitro* with β-glucans from Shiitake showed increased levels of the pro-inflammatory cytokines IL1-β, IL-8, NF-κβ. However, in contrast to our results THP1 stimulated with β-glucans showed an increased IL-10 expression [24]. Whether increased IL-10 hinders regulation of the immune response or alternatively is a transitory effect is not known. *In vivo* fungal β-glucans mostly elicit anti-inflammatory, anti-oxidant and anti-tumour effects [25]. Lentinan from shiitake can regulate MAP kinases JNK1/2 and ERK1/2 and enhance the nuclear translocation of NF-κB but without NO and TNF-α production in RAW 264.7 cells [26,27].

β-glucans from shiitake can augment phagocytosis, ConA-induced splenocyte proliferation, DTH reactions, TNF-α and IFN-γ production and finally NO production in peritoneal macrophages, that might be underlying mechanisms explaining their anti-tumour effects [3]. These apparent controversial results can be better explained with data obtained in this study. After dietary intake of β-glucans from *Lentinula edodes* macrophages clearly downregulate IL-10 expression which is indicative of immune stimulation, together with IL-4 and IFN-γ induced expression clearly suggesting activation of pro-inflammatory but also anti-inflammatory pathways. This can be achieved by the presence of M1 and M2 differentiated macrophages in the periphery or by the induction of these cytokines in the same cell. In any case, the response to β-glucans involves activation of both subpopulations likely leading to an overall activation state useful in the fight against a broad spectrum of pathogens [28] and avoiding the exacerbation of the inflammatory response.

However, care should be taken when evaluating mRNA relative quantification due to potential post-transcriptional modifications that may impair the conclusions. Protein production was evaluated here by quantifying cytokines in BDM and lymphocyte supernatants confirming results at the protein level, however, proteomic analysis or macrophage migration assays may depict insight mechanisms rendering the picture clearer.

Further studies will elucidate whether the immune response elicited by a β-glucan enriched diet is strong and durable enough to be considered as an alternative therapeutic tool allowing antibiotic elimination.
